# Regenerative Capacity of Macrophages for Remyelination

**DOI:** 10.3389/fcell.2016.00047

**Published:** 2016-05-20

**Authors:** Khalil S. Rawji, Manoj K. Mishra, V. Wee Yong

**Affiliations:** Hotchkiss Brain Institute and the Department of Clinical Neurosciences, University of CalgaryCalgary, AB, Canada

**Keywords:** microglia, macrophage, remyelination, oligodendrocyte, myelin

## Abstract

White matter injury, consisting of loss of axons, myelin, and oligodendrocytes, is common in many neurological disorders and is believed to underlie several motor and sensory deficits. Remyelination is the process in which the insulative myelin sheath is restored to axons, thereby facilitating recovery from functional loss. Remyelination proceeds with oligodendrocyte precursor cells (OPCs) that differentiate into oligodendrocytes to synthesize the new myelin sheath after demyelination. This process is influenced by several factors, including trophic factors, inhibitory molecules in the lesion microenvironment, age of the subject, as well as the inflammatory response. Currently studied strategies that enhance remyelination consist of pharmacological approaches that directly induce OPC differentiation or using agents to neutralize the inhibitory microenvironment. Another strategy is to harness a reparative inflammatory response. This response, coordinated by central nervous system resident microglia and peripherally-derived infiltrating macrophages, has been shown to be important in the remyelination process. These innate immune cells perform important functions in remyelination, including the proteolysis and phagocytosis of inhibitory molecules present in the lesion microenvironment, the provision of trophic and metabolic factors to OPCs, in addition to iron handling capacity. Additionally, an initial pro-inflammatory phase followed by a regulatory/anti-inflammatory phase has been shown to be important for OPC proliferation and differentiation, respectively. This review will discuss the beneficial roles of macrophages/microglia in remyelination and discuss therapeutic strategies to obtain the optimal regenerative macrophage phenotype for enhanced remyelination.

## Introduction

Many neurological conditions exhibit white matter injury, where substantial damage to oligodendrocytes and the protective myelin sheath is observed (Matute, 2010). This lipid-rich myelin sheath is produced by oligodendrocytes in the central nervous system (CNS) to insulate axons, provide metabolic support, and facilitate rapid signal transduction (Franklin and Ffrench-Constant, 2008; Saab et al., 2013). Restoration of the myelin sheath after a demyelinating insult is an endogenous process termed remyelination, and is important for re-establishing rapid signal conduction and metabolic support to the axon (Smith et al., 1979, 1981; Lee et al., 2012). After a demyelinating injury, oligodendrocyte precursor cells (OPCs), which reside throughout the gray and white matter of the CNS, become activated and subsequently proliferate and migrate to the lesion site (Kotter et al., 2011). Upon cues from the microenvironment, OPCs contact denuded axons, activate transcriptional programs for myelin gene expression, and differentiate into myelinating oligodendrocytes. Mature oligodendrocytes wrap cytoplasmic processes around the denuded axon, forming the newly synthesized myelin sheath.

Animal models demonstrate robust remyelination after injury and there is evidence to suggest that this process occurs in humans as well (Patrikios et al., 2006; Franklin and Ffrench-Constant, 2008). There is also evidence, however, to suggest that many demyelinated plaques in the human CNS do not remyelinate (Wolswijk, 1998; Chang et al., 2002). There may be several reasons as to why remyelination fails in these lesions, including inhibitors that are present in the lesion microenvironment, age of the subject, and the inflammatory response. To date, several inhibitors of remyelination have been identified, and these include the chondroitin sulfate proteoglycans (CSPGs), hyaluronan, myelin debris, semaphorin 3A, and LINGO-1 (Keough and Yong, 2013). When such molecules are neutralized in animal models, remyelination is enhanced. It is also known through both rodent and human studies that remyelination decreases with age (Sim et al., 2002; Zhao et al., 2006; Shen et al., 2008; Goldschmidt et al., 2009; Ruckh et al., 2012). Aging animals receiving a demyelination insult demonstrate delayed remyelination, associated with decreased OPC recruitment and differentiation (Gilson and Blakemore, 1993; Shields et al., 1999; Sim et al., 2002). Another factor contributing to the remyelination process is the innate inflammatory response, predominantly consisting of macrophages and microglia (Miron and Franklin, 2014). Studies that deplete these cells following injury report impaired remyelination, supporting the important role of these cells in this process (Kotter et al., 2001, 2005). This review will discuss the roles of macrophages and microglia in remyelination and suggest potential therapeutic strategies to promote a regenerative macrophage phenotype for remyelination.

## Microglia, macrophages, and remyelination

Murine studies have demonstrated that microglia and other tissue resident macrophages arise from erythromyeloid precursors in the embryonic yolk sac, which then migrate to the developing tissues to establish self-renewing populations throughout adulthood (Ajami et al., 2007; Ginhoux et al., 2010; Schulz et al., 2012; Kierdorf et al., 2013; Bruttger et al., 2015). In the healthy adult CNS, microglia are ramified in appearance, extending and retracting processes to survey the local microenvironment (Nimmerjahn et al., 2005). Upon injury to the CNS, these cells become activated and amoeboid in appearance, releasing many pro- and anti-inflammatory cytokines and chemokines as an innate defense mechanism against pathogens. Consequently, such a response is frequently toxic to various neural cells. Despite this destructive immune response, certain cytokines and growth factors, in addition to the phagocytosis and removal of inhibitory molecules, aids in the resolution and repair of injury. Tissue-infiltrating macrophages responding to injury derive from circulating monocytes which have rapid turnover and are continuously produced by hematopoietic stem cells in the bone marrow (Prinz and Priller, 2014). Upon injury, monocytes respond to chemotactic factors and migrate to the injury site, differentiating into amoeboid macrophages and secreting an array of immune molecules which are toxic but also aid in the repair of the lesion site. Due to the phenotypic similarity of activated microglia and infiltrating macrophages, it has been difficult by histology to distinguish these cells and their respective functions in the lesion microenvironment. As such, these cells have commonly been termed macrophages/microglia. Despite this difficulty in discerning these two cell types, progress has been made in assigning a unique genetic signature to microglia. Several microglia-specific genes have now been identified, including *tmem119, fcrls*, and *p2ry12* (Butovsky et al., 2014). In addition, a novel transgenic approach has been employed to study the differential roles of microglia and infiltrating monocytes in experimental autoimmune encephalomyelitis. This technique takes advantage of microglia and monocytes expressing different levels of CX3CR1 and CCR2 (Yamasaki et al., 2014).

Macrophages and microglia demonstrate a diverse array of phenotypic signatures that are dependent on the tissue microenvironment (Gosselin et al., 2014; Lavin et al., 2014). As such, these cells display a broad spectrum of activation ranging from pro-inflammatory to regulatory/anti-inflammatory activities. Previously, this spectrum was simply classified dichotomously as “M1” pro-inflammatory macrophages and “M2” regulatory/anti-inflammatory macrophages (Martinez et al., 2008). It has been recently appreciated, however, that a spectrum model of polarization may be better able to describe the diverse plasticity these cells demonstrate (Xue et al., 2014). Interestingly, this study showed that human macrophages *in vitro* may adopt at least nine different broadly categorized activation states depending on the induction stimuli presented. To further support this spectrum model of polarization, another study showed that the majority of activated macrophages/microglia in human MS lesions possess phenotypic markers of both pro-inflammatory and regulatory macrophages (Vogel et al., 2013). Due to this vast array of macrophage plasticity, it has been proposed to classify macrophage polarization based on a defined set of parameters, such as species and strain used, activating stimuli, and tissue-culture conditions (Murray et al., 2014).

Macrophages and microglia can be neurotoxic through the release of pro-inflammatory cytokines, reactive oxygen species, and through the reactivation of encephalitogenic T cells (Rawji and Yong, 2013). Several studies have demonstrated that activated macrophages and microglia can be toxic to oligodendrocytes and neurons (Merrill and Zimmerman, 1991; Merrill et al., 1993; Kigerl et al., 2009; Moore et al., 2015). Additionally, studies in which microglia or macrophages are depleted report an improvement in the severity of experimental autoimmune encephalomyelitis (Martiney et al., 1998; Tran et al., 1998; Heppner et al., 2005). Despite these detrimental aspects, studies have shown that these cells are also necessary for remyelination (Rawji and Yong, 2013). Depletion of macrophages/microglia in focal demyelination models impairs remyelination, associated with a deficit in the clearance of inhibitory myelin debris (Kotter et al., 2001, 2005, 2006). Interestingly, remyelination is impaired only when these cells are depleted within the first 8 days following demyelination, suggesting that the early activity of these cells is important for effective remyelination (Kotter et al., 2001). Complementing this finding is another study in which minocycline was used to inhibit macrophages/microglia within the first 3 days following focal demyelination of the rat caudal cerebellar peduncle (Li et al., 2005). When macrophages/microglia were inhibited with minocycline, the OPC response and remyelination was impaired. It has been reported that, at an early time point post-demyelination, a predominance of pro-inflammatory macrophages/microglia are present, and depletion of these cells impairs OPC proliferation (Miron et al., 2013). At a later time point, regulatory macrophages/microglia predominate, and depletion of these cells impairs OPC differentiation (Miron et al., 2013). In addition, this group observed the increased presence of regulatory macrophages/microglia in acute active and the rim of chronic active human MS lesions, areas in which remyelination may be ongoing. Complementing these depletion studies are studies in which macrophages/microglia are stimulated, resulting in enhanced remyelination (Glezer et al., 2006; Setzu et al., 2006; Doring et al., 2015). Injection of regulatory microglia stimulated with interleukin-4 into the cerebrospinal fluid of rodents with experimental autoimmune encephalomyelitis increased oligodendrogenesis in the spinal cord (Butovsky et al., 2006). In addition to macrophages/microglia being important for remyelination, it has also been shown that infiltration of myelin-specific T cells can enhance oligodendrogenesis in the mouse dentate gyrus (Hvilsted Nielsen et al., 2011).

In support of the contention that macrophages/microglia can be beneficial for remyelination in human pathologies such as MS, it has been noted that the density of O4-positive OPCs across several MS spinal cord and brain specimens correlated with the density of phase-bright, debris-laden macrophages (Wolswijk, 2002). The elevated density of HLA-DR positive macrophages/microglia at the lesion border of MS plaques correlated with more prominent remyelination (Patani et al., 2007). Though these studies suggest that inflammation may be beneficial for remyelination in human MS lesions, several studies have demonstrated the opposite, whereby an increased density of macrophages/microglia is correlated with demyelination and acute axonal injury (Ferguson et al., 1997; Lucchinetti et al., 1999; Kuhlmann et al., 2002). Additionally, a decrease in the macrophage inhibitory molecules CD200 and CD47 has been documented in chronic active and inactive MS lesions, suggesting an elevated pro-inflammatory phenotype of macrophages/microglia in these lesions, possibly contributing to increased neurotoxicity (Koning et al., 2007).

**Figure 1:**
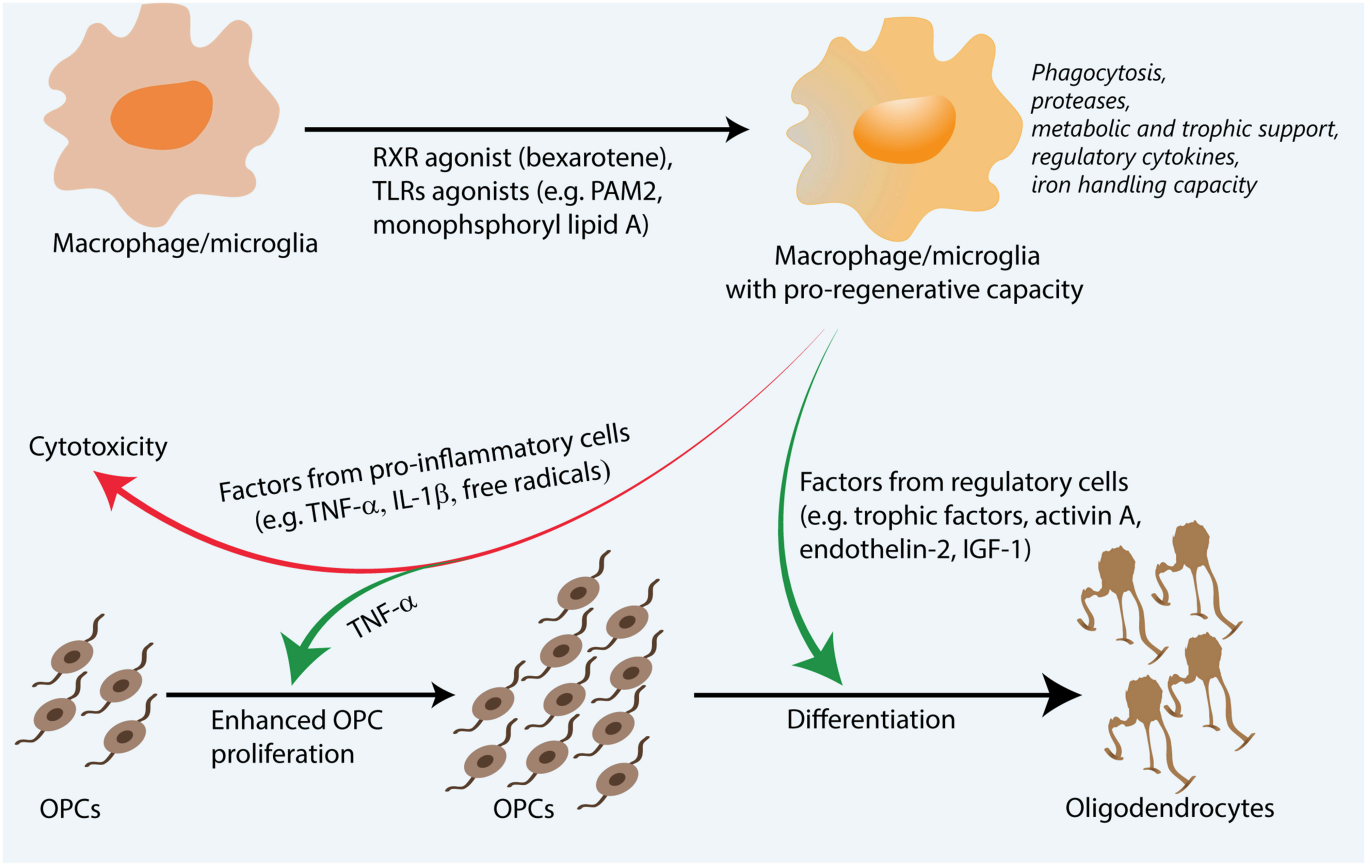
**Regenerative macrophages for remyelination**. Following their stimulation with agents such as Toll-like receptor (TLR) ligands, retinoid X receptor (RXR) agonists, both pro-inflammatory and regulatory factors are necessary for efficient remyelination. Though pro-inflammatory factors can be toxic to oligodendrocyte precursor cells (OPCs), they are also thought to enhance OPC proliferation. Regulatory factors such as activin A and insulin-like growth factor 1 (IGF-1) are thought to promote OPC differentiation. Additionally, functions such as proteolysis and phagocytosis of inhibitory myelin debris and extracellular matrix molecules, metabolic support through lactate production, as well as iron handling capacity also influence remyelination. Pharmacological approaches that are able to synergistically enhance many of these processes are promising for remyelination and complement existing strategies to directly induce OPC differentiation or neutralize the inhibitory microenvironment.

Some of the factors underlying the regenerative properties of macrophages/microglia in remyelination have been elucidated not only through remyelination models, but also in models of spinal cord injury, Alzheimer's disease, and optic crush (Shechter and Schwartz, 2013). Such factors include the phagocytic removal of inhibitory molecules, the remodeling of the extracellular matrix, the release of growth factors and metabolites important for OPC maturation, as well as the storage and release of different forms of iron to minimize toxicity (Figure 1). As mentioned above, myelin debris is inhibitory for OPC differentiation and axon regeneration (Filbin, 2003; Kotter et al., 2006). Depletion of macrophages/microglia results in accumulation of myelin debris and impaired remyelination, suggesting that these cells are important for the phagocytic clearance of this debris (Kotter et al., 2005).

Macrophages/microglia are also thought to play an important role in the remodeling of the extracellular matrix. After injury to the CNS, molecules comprising components of the extracellular matrix are abundantly secreted, including the CSPGs, which are known to impair oligodendrocyte differentiation (Siebert and Osterhout, 2011) and to inhibit remyelination (Lau et al., 2012). Pharmacological approaches that inhibit the synthesis of these inhibitory extracellular matrix molecules have enhanced remyelination (Lau et al., 2012; Keough et al., 2016). Macrophages/microglia are thought to be important in degrading these inhibitory molecules, allowing the microenvironment to be more conducive for remyelination. These cells release a variety of proteases which are capable of cleaving the inhibitory substrates (Gibbs et al., 1999). In mice genetically deficient for matrix metalloproteinase-9, there is an accumulation of CSPGs associated with a decrease in remyelination (Larsen et al., 2003).

Additionally, several studies have demonstrated that macrophages/microglia are capable of releasing important growth factors and metabolites for axon regeneration and OPC maturation. For example, oncomodulin produced from macrophages was shown to enhance axon regeneration after optic nerve crush (Yin et al., 2006). Growth factors released by macrophages/microglia that have been shown to be important for OPC proliferation or differentiation include insulin-like growth factor 1, activin-A, endothelin-2, heparin-binding epidermal growth factor, platelet derived growth factor, and fibroblast growth factor (McMorris et al., 1986; Higashiyama et al., 1991; Armstrong et al., 2002; O'Donnell et al., 2002; Woodruff et al., 2004; Ruckh et al., 2012; Miron et al., 2013; Yuen et al., 2013; Scafidi et al., 2014).

In addition to growth factors, it is thought that pro-inflammatory cytokines produced by activated macrophages/microglia are essential for myelin repair. Studies in which tumor necrosis factor-α or interleukin-1β are depleted report that remyelination is impaired, suggesting that these cytokines are important for remyelination (Arnett et al., 2001; Mason et al., 2001). Moore et al. (2015) showed that cytokines produced by human macrophages and microglia can act directly on OPCs or indirectly through astrocytes. Though tumor necrosis factor-α is thought to be important for remyelination, this group documented this cytokine to be cytotoxic to OPCs (Moore et al., 2015) Additionally, macrophages elicit unique metabolic signatures dependent on the microenvironment (Jha et al., 2015). Recent work has suggested that murine macrophages activated with lipopolysaccharide employ glycolysis rather than oxidative phosphorylation for energy production, producing lactate as a by-product (Jha et al., 2015). As it was shown that lactate is important for oligodendrogenesis (Rinholm et al., 2011), it is conceivable that pro-inflammatory macrophages responding to demyelination may be providing lactate as a metabolic factor supporting remyelination.

The role of macrophages is multifaceted and complex in iron homeostasis. Iron can be potentially damaging through free radical-mediated toxicity but iron is also required as a co-factor for many enzymes that regulate metabolism, proliferation, and differentiation of cells of the oligodendrocyte lineage, and the deposition of myelin membranes (Stephenson et al., 2014). Iron handling is a function that is conserved in both rodent and human polarized macrophages (Corna et al., 2010; Recalcati et al., 2010). Macrophage polarization differentially affects the expression of ferritin and ferroportin, affecting the capacity of macrophages to store and release iron, respectively. Pro-inflammatory macrophages express genes that favor the sequestration of iron, whereas regulatory macrophages express genes that promote iron release (Recalcati et al., 2010). It was shown in experimental autoimmune encephalomyelitis that the ability of macrophages/microglia to handle iron is disrupted. In this study, macrophages/microglia demonstrated a defect in iron efflux, resulting in the gradual accumulation of iron as the disease progressed (Zarruk et al., 2015). Another study conducted by this group showed that increased loading of iron in macrophages induces a pro-inflammatory phenotype which may be detrimental to repair (Kroner et al., 2014). Despite the potential for macrophages/microglia to exacerbate iron toxicity, it is known that oligodendrocytes require iron for their growth and differentiation (Schonberg and McTigue, 2009). When iron was chelated, there was a reduction in the generation of new oligodendrocytes (Schonberg and McTigue, 2009). The same group subsequently showed that activated macrophages are able to transfer ferritin, the iron storage protein, to OPCs, enhancing their differentiation into oligodendrocytes (Schonberg et al., 2012). Thus, the role of macrophages in iron homeostasis is, indeed, very complex and much work is necessary to determine how best to harness the pro-regenerative capacity of macrophages in the context of iron handling.

## Effect of aging on macrophages, microglia, and remyelination

It has been well documented that, as with other regenerative processes, remyelination is delayed with aging. Aging rodents receiving focal demyelination remyelinate slower than young rodents (Gilson and Blakemore, 1993; Shields et al., 1999; Zhao et al., 2006; Ruckh et al., 2012). This lag in remyelination is associated with a delay in the recruitment and differentiation of OPCs, in addition to a dysregulation in several growth factors implicated in OPC recruitment and differentiation (Sim et al., 2002). It is now appreciated that this age-related impairment in remyelination is associated with a senescent macrophage/microglia response (Zhao et al., 2006; Ruckh et al., 2012). In aging rodents receiving focal demyelination, there is a delay in the recruitment of macrophages/microglia associated with an alteration in several inflammatory cytokines and chemokines (Zhao et al., 2006). In addition, there is an accumulation of inhibitory myelin debris, suggesting that phagocytosis is impaired in these cells (Ruckh et al., 2012). Indeed, several studies have reported impaired phagocytosis in aging macrophages and microglia (Rawji et al., 2016). In the context of myelin phagocytosis, a deficiency in retinoid X receptor alpha signaling has been attributed (Natrajan et al., 2015). Aging macrophages and microglia also demonstrate a dysregulation in cytokine secretion, with aging macrophages exhibiting defective cytokine secretion, whereas microglia display enhanced pro-inflammatory cytokine secretion (Rawji et al., 2016). When a young mouse is parabiotically paired to an aging mouse receiving focal demyelination, remyelination is rejuvenated (Ruckh et al., 2012). This study showed that rejuvenation of remyelination is partially due to young green fluorescent protein-tagged macrophages present in the aging lesion and partially due to humoral factors in the young circulation, providing proof-of-principle that the aging microenvironment can be partially overcome by young macrophages. Pharmacological approaches which are able to reverse macrophage/microglia senescence would be promising in enhancing remyelination in aging subjects.

## Promoting regenerative macrophages and remyelination

There have been many studies recently published that have described pharmacological strategies to promote remyelination. Many of these studies have screened clinically-approved medications *in vitro* to identify candidates which can directly act on OPCs to enhance their proliferation or differentiation. Some of these candidates have also been shown to enhance remyelination in animal models (Deshmukh et al., 2013; Mei et al., 2014; Najm et al., 2015). One such medication, clemastine, is currently being investigated in a clinical trial (Clinicaltrials.gov identifier NCT02040298). As pharmacologically stimulated OPCs still have to overcome an inhibitory microenvironment, other approaches have been made to neutralize or reduce the synthesis of inhibitors present in the lesion (Mi et al., 2009; Lau et al., 2012; Keough et al., 2016). One such inhibitor is LINGO-1, of which a neutralizing antibody to this protein is currently being studied in a phase 2 clinical trial (Clinicaltrials.gov identifier NCT01864148). Harnessing the regenerative capacity of macrophages/microglia represents another strategy which can both enhance the activity of OPCs through metabolic and trophic support as well as neutralize the inhibitory microenvironment through protease activity and phagocytosis (Figure 1). Thus, strategies which enhance a regenerative macrophage/microglia phenotype are promising for remyelination. Such a direction should act in combination with approaches that directly promote OPC differentiation, or with strategies that counter the inhibitory microenvironment. This section will discuss several therapeutic strategies which enhance regenerative aspects of macrophages/microglia.

One of the major functions of macrophages and activated microglia is phagocytosis. As discussed, the lesion microenvironment contains many inhibitory molecules, including myelin debris and inhibitory components of the astrogliotic scar, such as CSPGs. Therapies which act to enhance phagocytosis are promising for remyelination. The clinically approved retinoid X receptor agonist, bexarotene, was shown to enhance phagocytosis in monocytes derived from multiple sclerosis patients (Natrajan et al., 2015). Such an agent may be effective in improving the clearance of inhibitory molecules in the lesion microenvironment. Other agents that may aid in the clearance of myelin debris are protollin and monophosphoryl lipid A (Frenkel et al., 2013; Michaud et al., 2013). Though both these medications were studied in the context of amyloid-β removal, it is conceivable that they would be also effective in the clearance of myelin debris. As molecules such as CSPGs likely need to be proteolytically degraded before being phagocytosed, agents that increase the secretion of proteases, in addition to phagocytosis, would presumably enhance the clearance of these inhibitory components. As monophosphoryl lipid A is a Toll-like receptor 4 agonist, and that stimulation of this receptor is known to enhance protease secretion (Gibbs et al., 1999), this agent is a promising candidate for enhancing both the phagocytic and extracellular matrix degrading capacities of macrophages. Both activities seem beneficial for regeneration.

As pro-inflammatory macrophages may be neurotoxic through the secretion of excess free radicals and pro-inflammatory cytokines, strategies which promote a regulatory macrophage phenotype appear promising. Regulatory macrophages are thought to produce important growth factors for OPC differentiation, such as activin-A (Miron et al., 2013). Agents which have been shown to modulate macrophage polarization toward a regulatory phenotype include interferon-β1 and the peroxisome proliferator-activated receptor α/γ agonist, DSP-8658 (Yamanaka et al., 2012; Cohen et al., 2014). Despite the promise of such a strategy, however, it is now appreciated that remyelination requires both an initial pro-inflammatory macrophage/microglia response with a subsequent regulatory response (Miron et al., 2013). This study showed that the initial pro-inflammatory response contributed to OPC proliferation, whereas the subsequent regulatory response aided in OPC differentiation (Miron et al., 2013). Therefore, strategies that are able to amplify a regulatory phenotype with a mild pro-inflammatory signature would seem to hold greater promise for enhancing both OPC proliferation and differentiation. Such a treatment may involve the combinational treatment with amphotericin B and macrophage colony-stimulating factor, which was shown to enhance OPC recruitment and remyelination by activating both pro-inflammatory and regulatory macrophage/microglia signatures (Doring et al., 2015); the toxicity of amphotericin B, however, may preclude its use as a remyelination agent.

In conclusion, therapeutic strategies which are able to harness both the pro-inflammatory and regulatory signatures required from remyelination, taking into account when these treatments are required temporally following an injury, would likely result in the optimal regenerative macrophage phenotype (Figure 1). However, such approaches must take into account the potential that an excessively stimulated macrophage/microglia can also elaborate toxic factors that would be counterproductive to enhancing repair. Further research investigating the immense transcriptional regulation of macrophages may yield information as to which combinations of microenvironmental stimuli produce the delicate balance of pro-inflammatory and regulatory outputs required for efficient remyelination.

## Conclusion

Injury to the white matter occurs in many neurological conditions and results in damage to oligodendrocytes and the insulative myelin sheath. Though remyelination can occur through the recruitment and differentiation of endogenously present OPCs, there are many instances in which remyelination fails, resulting in progressive axonal loss, and clinical disability. Factors underlying the impairment in remyelination include inhibitors present in the microenvironment, dysregulation of growth factors important for OPC recruitment and differentiation, aging, as well as the innate inflammatory response consisting of macrophages and microglia. Studies in which these cells are depleted report an impairment in remyelination, emphasizing the important role these cells have in clearing inhibitory debris, producing growth factors, and providing metabolic support. Importantly, these cells become dysregulated with aging, and it is this senescence that is thought to influence the age-related impairment in remyelination. It is now known that remyelination is efficient when there is a balance and temporal sequence of both pro-inflammatory and regulatory macrophages/microglia. Therapeutic strategies which harness this balance of pro-inflammatory and regulatory features, in addition to promoting phagocytosis, extracellular matrix remodeling, and limiting excessive and harmful pro-inflammatory cytokine secretion, will conceivably be beneficial for remyelination.

## Author contributions

All authors listed, have made substantial, direct and intellectual contribution to the work, and approved it for publication.

## Funding

We thank the Canadian Institutes of Health Research, the Multiple Sclerosis Society of Canada and the Alberta Innovates—Health Solutions CRIO Team program for support of operating funds. KR is supported by a Vanier Canada Graduate Scholarship and a studentship from the University of Calgary Faculty of Medicine. MM was supported by a fellowship from Alberta Innovates—Health Solutions. VW acknowledge salary support from the Canada Research Chair (Tier 1) program.

### Conflict of interest statement

The authors declare that the research was conducted in the absence of any commercial or financial relationships that could be construed as a potential conflict of interest.
